# Racial and Ethnic Disparities in Prostate Cancer Outcomes in the Veterans Affairs Health Care System

**DOI:** 10.1001/jamanetworkopen.2021.44027

**Published:** 2022-01-18

**Authors:** Kosj Yamoah, Kyung Min Lee, Shivanshu Awasthi, Patrick R. Alba, Cristina Perez, Tori R. Anglin-Foote, Brian Robison, Anthony Gao, Scott L. DuVall, Evangelia Katsoulakis, Yu-Ning Wong, Sarah C. Markt, Brent S. Rose, Ryan Burri, Carrie Wang, Okoduwa Aboiralor, Angelina K. Fink, Nicholas G. Nickols, Julie A. Lynch, Isla P. Garraway

**Affiliations:** 1 H. Lee Moffitt Cancer Center & Research Institute, Tampa, Florida; 2 Department of Veteran Affairs Salt Lake City Health Care System, Salt Lake City, Utah; 3 James A. Haley Veterans Hospital, Tampa, Florida; 4 Pearlman School of Medicine, University of Pennsylvania, Philadelphia; 5 Department of Population and Quantitative Health Science, Case Western Reserve University, Cleveland, Ohio; 6 Department of Radiation Medicine and Applied Sciences, University of California, San Diego, San Diego; 7 Bay Pines VA Healthcare System, Tampa, Florida; 8 Morsani College of Medicine, University of South Florida, Tampa; 9 David Geffen School of Medicine at UCLA, University of California, Los Angeles, Los Angeles

## Abstract

**Importance:**

Prostate cancer (PCa) disproportionately affects African American men, but research evaluating the extent of racial and ethnic disparities across the PCa continuum in equal-access settings remains limited at the national level. The US Department of Veterans Affairs (VA) Veterans Hospital Administration health care system offers a setting of relatively equal access to care in which to assess racial and ethnic disparities in self-identified African American (or Black) veterans and White veterans.

**Objective:**

To determine the extent of racial and ethnic disparities in the incidence of PCa, clinical stage, and outcomes between African American patients and White patients who received a diagnosis or were treated at a VA hospital.

**Design, Setting, and Participants:**

This retrospective cohort study included 7 889 984 veterans undergoing routine care in VA hospitals nationwide from 2005 through 2019 (incidence cohort). The age-adjusted incidence of localized and de novo metastatic PCa was estimated. Treatment response was evaluated, and PCa-specific outcomes were compared between African American veterans and White veterans. Residual disparity in PCa outcome, defined as the leftover racial and ethnic disparity in the outcomes despite equal response to treatment, was estimated.

**Exposures:**

Self-identified African American (or Black) and White race and ethnicity.

**Main Outcomes and Measures:**

Time to distant metastasis following PCa diagnosis was the primary outcome. Descriptive analyses were used to compare baseline demographics and clinic characteristics. Multivariable logistic regression was used to evaluate race and ethnicity association with pretreatment clinical variables. Multivariable Cox regression was used to estimate the risk of metastasis.

**Results:**

Data from 7 889 984 veterans from the incidence cohort were used to estimate incidence, whereas data from 92 269 veterans with localized PCa were used to assess treatment response. Among 92 269 veterans, African American men (n = 28 802 [31%]) were younger (median [IQR], 63 [58-68] vs 65 [62-71] years) and had higher prostate-specific antigen levels (>20 ng/mL) at the time of diagnosis compared with White men (n = 63 467; [69%]). Consistent with US population-level data, African American veterans displayed a nearly 2-fold greater incidence of localized and de novo metastatic PCa compared with White men across VA centers nationwide. Among veterans screened for PCa, African American men had a 29% increased risk of PCa detection on a diagnostic prostate biopsy compared with White (hazard ratio, 1.29; 95% CI, 1.27-1.31; *P* < .001). African American men who received definitive primary treatment of PCa experienced a lower risk of metastasis (hazard ratio, 0.89; 95% CI, 0.83-0.95; *P* < .001). However, African American men who were classified as “other” race and received treatment were more likely to develop metastasis (adjusted hazard ratio, 1.29; 95% CI, 1.17-1.42; *P* < .001). Using the actual rate of metastasis from veterans who received definitive primary treatment, a persistent residual metastatic burden for African American men was observed across all National Comprehensive Cancer Network risk groups (low risk, 4 vs 2 per 100 000; intermediate risk, 13 vs 6 per 100 000; high risk, 19 vs 9 per 100 000).

**Conclusions and Relevance:**

This cohort analysis found significant disparities in the incidence of localized and metastatic PCa between African American veterans and White veterans. This increased incidence is a major factor associated with the residual disparity in PCa metastasis observed in African American veterans compared with White veterans despite their nearly equal response to treatment.

## Key Points

**Question:**

Are there racial and ethnic disparities associated with the incidence, clinical stage, and outcomes of prostate cancer among men treated in the Veterans Affairs health care system?

**Findings:**

In this cohort study of 7.8 million patients, compared with White veterans, African American (or Black) veterans displayed increased risk of developing localized and de novo metastatic prostate cancer across nationwide Veterans Affairs centers. Despite equitable treatment response, a persistent residual metastatic burden of nearly 2-fold was observed for African American veterans across National Comprehensive Cancer Network risk groups, when factoring in the higher incidence rate.

**Meaning:**

Treatment in an equal-access setting may partially address institution-specific disparities, but overall racial disparity gaps remain.

## Teaser

This cohort study assesses the extent of racial and ethnic disparities in the incidence, clinical stage, and outcomes of prostate cancer among African American and White men treated in the Veterans Affairs health care system.

## Introduction

Men living in the United States who self-identify as African American (or as Black but hereinafter referred to as African American) historically experience an increased burden of prostate cancer (PCa) in terms of incidence of both localized and metastatic disease as well as morbidity and mortality.^1,2^ Specifically, compared with White men, African American men are more likely to receive a diagnosis of PCa and are nearly 2 times more likely to die of PCa.^1^ Although the incidence rates of PCa vary from state to state, racial and ethnic disparities are consistently shown across most geographical regions in the US.^1^ A major component of these disparities may be explained by socioeconomic status, which reflects access to care.^3^ However, differences in access to care are insufficient to explain the increased incidence of PCa among African American men.^4^ Several recent studies have invigorated the debate about whether equal access to care is sufficient to eliminate some, if not all, of the observed racial and ethnic disparities.^5,6,7,8^ Although access to quality care is an essential component for reducing disparities in disease outcomes, it is important to consider that the racial and ethnic disparities observed in PCa are multidimensional and likely span the disease continuum, including incidence, stage at diagnosis, and frequency of adverse pathological features.^1,9,10^ In addition, the use of overall survival end points in the evaluation of PCa disparities often does not capture the full spectrum of PCa morbidity in the African American population^11^ because length of life does not consider quality of life, which is frequently adversely affected by disease and treatment.^12^ However, distant metastasis, which is strongly correlated with PCa morbidity and mortality, may represent a meaningful variable to capture the broader burden of PCa for African American men vs White men, in addition to overall survival.^11,13^

The US Department of Veterans Affairs (VA) health care system serves approximately 20 million US veterans and is the largest integrated health care system for cancer care in the US. By providing high-quality care to veterans regardless of race and ethnicity, sex, geographic location, or economic circumstance, the VA is considered an equal-access system compared with other large health care systems and thus provides a unique environment to investigate PCa health disparities across the disease continuum.^14,15^ In this cohort analysis, we quantified incidence-level disparities between African American veterans and White veterans nationwide and assessed treatment response by the presence of distant metastasis. More importantly, we evaluated the presence of residual disparity, which is defined as the leftover racial and ethnic disparity in the outcomes after treatment.

## Methods

### Data Sources and Study Population

**Figure 1.**
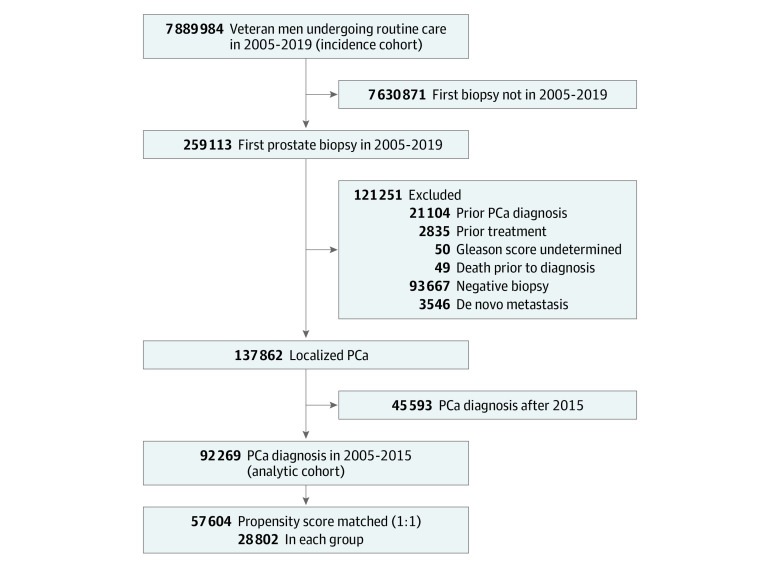
Study Flow Diagram PCa indicates prostate cancer.

This retrospective observational cohort analysis was conducted using data from the VA national electronic health record in the Corporate Data Warehouse and the VA Central Cancer Registry, accessed through the VA Informatics and Computing Infrastructure. The Corporate Data Warehouse contains patient demographic, clinical, and treatment data as well as outcome information. To perform a comprehensive analysis of PCa incidence and de novo metastasis burden at the time of diagnosis, we created an incidence cohort of 7 889 984 African American veterans and White veterans undergoing routine or PCa-related care at 135 VA medical centers nationwide from 2005 to 2019. Routine care was defined as having 2 or more clinical encounters (inpatient or outpatient), regardless of PCa. The PCa-related care included urology clinic visit, prostate-specific antigen (PSA) test, prostate biopsy, and encounters associated with receiving a PCa diagnosis. We identified PSA tests using a combination of current procedural terminology codes, logical Observation Identifiers Names and Codes, and laboratory test names. In the incidence cohort, we identified a PSA screening cohort of 2 788 003 veterans who underwent PSA screening at the VA. To investigate clinical presentation and outcomes, the final analytic cohort of veterans with localized PCa (M0 cohort) was limited to 92 269 patients who received a diagnosis of PCa between 2005 and 2015, which enabled at least 5 years of follow-up (Figure 1). This retrospective study followed the Strengthening the Reporting of Observational Studies in Epidemiology (STROBE) reporting guideline.^16^ This study was approved by the Salt Lake City Institutional Review Board and Research & Development Committee. Analyses were conducted with an approved waiver for obtaining informed consent and with Health Insurance Portability and Accountability Act of 1996 authorization.

We used a validated natural language processing (NLP) tool to identify the presence of metastatic PCa (mPCa) and extracted the date of the first clinical encounter associated with the mPCa diagnosis.^17^ We also used NLP to extract the Gleason score from surgical pathology reports and to confirm PCa diagnoses. The NLP tools were applied to all clinical notes from urology, oncology, pathology, radiation oncology, radiology, and chemotherapy infusion. Patients with de novo mPCa were defined as having an NLP-identified diagnosis of mPCa within 90 days of receiving their initial PCa diagnosis. Both the structured algorithm and the NLP tools were validated by comparing the output to the cancer registry.

### Study Variables and End Points

To evaluate regional differences in the incidence of localized PCa and de novo mPCa between African American veterans and White veterans, we used the residential zip code linked to each case to examine incidence rates by county and state. Self-identified race and ethnicity were used to categorize patients as African American or White. White race and ethnicity included self-identified non-Hispanic White men, whereas African American included self-identified Black or African American Hispanic and non-Hispanic men. We obtained detailed information on patient baseline demographic and clinical characteristics, including age, pathological Gleason score, screening PSA levels, pretreatment PSA levels, time from diagnosis to definitive primary treatment (radiotherapy with or without androgen deprivation therapy and radical prostatectomy), and treatment status (definitive treatment vs “other”). Patients were assigned to National Comprehensive Cancer Network (NCCN) high-, intermediate-, and low-risk groups based on Gleason score, PSA level, and tumor stage.^18^ We calculated the time between the first PSA screening test and the date of the diagnostic prostate biopsy to test the difference in the rate of diagnosis between African American veterans and White veterans (PSA screening cohort; n = 2 788 003). To estimate the treatment response in the M0 cohort, the time to distant metastasis following PCa treatment was used as a primary end point (n = 92 269). The NLP tool was also applied to robustly identify the presence of metastasis after the diagnosis (index date), and the time of metastasis was estimated by subtracting the date of diagnosis from the date of metastasis. We also estimated PCa-specific mortality (PCSM) by using metastasis as proxy for PCa-specific death. Patients with metastasis who died during the follow-up were categorized as having a PCSM event, whereas patients who died with no documented metastasis were assigned death due to other causes.^11^ We used PCSM only to assess residual disparity; PCSM was not used in the survival analysis.

### Statistical Analysis

Using the total number of male veterans undergoing routine care as the denominator (N = 7 889 984; incidence cohort), we calculated annual, age-adjusted rates of PCa incidence and de novo mPCa by race and ethnicity. We used year 2000 US standard population weights to derive the yearly incidence rate per 100 000. To estimate the observed racial and ethnic disparity in the incidence of PCa across nationwide VA centers, we calculated the incidence of localized PCa and de novo mPCa among African American veterans relative to White veterans (eFigure 1 in the Supplement). To estimate the risk of PCa diagnosis after initial PSA screening among African American veterans and White veterans, we conducted a multivariable Cox proportional hazards model adjusted for PSA level, age at first PSA screening, and year of PSA screening and estimated the cumulative incidence of receiving a diagnostic prostate biopsy. To estimate the PSA-dependent risk of diagnostic prostate biopsy among African American veterans and White veterans, we further tested the interaction of race and ethnicity with PSA level and conducted a PSA level–stratified Cox model (<10 ng/mL, 10-20 ng/mL, >20 ng/mL [to convert to micrograms per liter, multiply by 1.0]).

Within the M0 cohort of 92 269 men, we performed descriptive analyses to compare baseline demographic and clinical characteristics. Among men diagnosed as having localized PCa (M0 cohort), we conducted multivariable logistic regression analysis to estimate the association of race and ethnicity with high-grade Gleason score (≥4 + 3 vs <4 + 3), pretreatment PSA level (>20 ng/mL vs ≤20 ng/mL), and high-risk disease (>20 ng/mL or Gleason score ≥8 or T category ≥cT3). Among the M0 cohort, we estimated the time to distant metastasis following PCa diagnosis using a treatment-stratified multivariable Cox proportional hazards model, adjusted for age at diagnosis, PSA level, Gleason score, time to treatment, use of androgen deprivation therapy, and diagnosis year. In a subgroup analysis, we performed propensity score matching to clinically balance the M0 cohort by matching the racial and ethnic groups in a ratio of 1:1 based on age, PSA level, and Gleason score. A subgroup analysis was conducted to validate the risk of metastasis in the Cox model. Finally, we estimated the residual disparity in metastatic burden and PCSM across NCCN risk groups between African American veterans and White veterans. For this, we derived the NCCN-stratified incidence rate of PCa using the incidence cohort and multiplied by the actual rate of metastasis and PCSM among patients who received definitive treatment within the VA hospitals. We performed 2-tailed hypothesis tests using a statistical significance level of 5%. All data were analyzed with SAS, version 9.4 (SAS Institute Inc).

## Results

Among 92 269 men diagnosed as having localized PCa (M0 cohort), African American men (n = 28 802 [31%]) were younger than White men (n = 63 467; [69%]) at diagnosis (median age, 63 years [IQR, 58-68 years] vs 65 years [IQR, 62-71 years]; *P* < .001). A higher proportion of African American men received cross-sectional abdomen/pelvis imaging (computed tomography or magnetic resonance imaging scans) within a year of their diagnosis (15 114 of 28 802 [52%] vs 30 002 of 63 467 [47%]; *P* < .001). We found no differences in the total number of prostate biopsies performed prior to the initial PCa diagnosis between African American men and White men (mean total number, 1.2 [range, 1.0-6.0] vs 1.2 [range, 1.0-7.0]; *P* = .50) (eTable 1 in the Supplement). Compared with White men, African American men were 4% more likely to have high-grade Gleason scores (≥4 + 3) (adjusted odds ratio, 1.04; 95% CI, 1.00-1.07), 92% more likely to have PSA levels higher than 20 ng/mL (adjusted odds ratio, 1.92; 95% CI, 1.82-2.02), and 21% more likely to be diagnosed as having high-risk PCa (adjusted odds ratio, 1.21; 95% CI, 1.16-1.25) (eFigure 2 in the Supplement).

### PCa Incidence in Equal-Access VA Health Care

**Figure 2.**
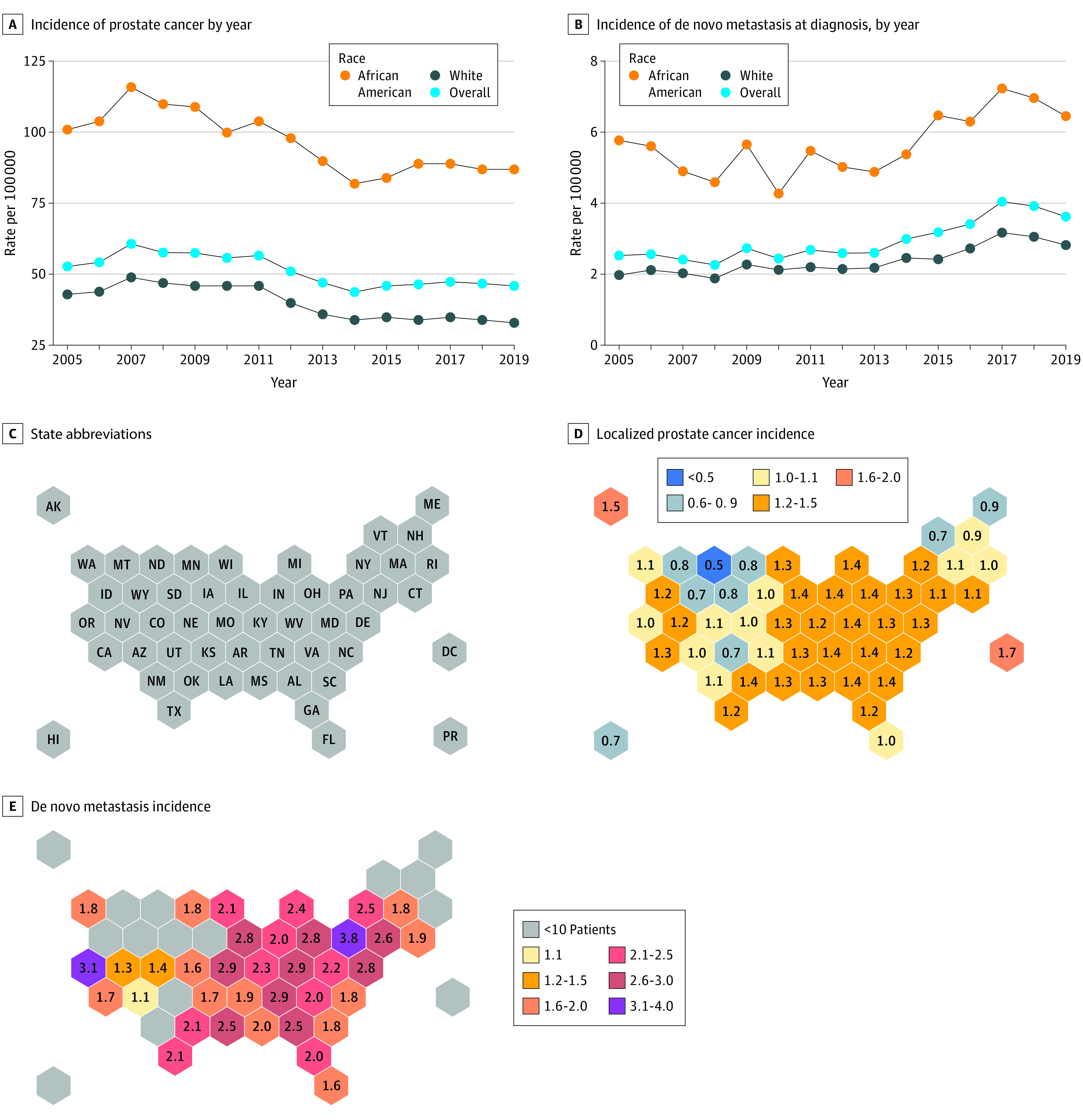
Incidence of Prostate Cancer and De Novo Metastasis at Diagnosis Across Veterans Affairs Centers C, Incidence-level racial and ethnic disparities across the US shown by state. Veteran Affairs–based racial and ethnic disparities in the incidence of prostate cancer (D) and de novo metastasis (E). Incidence rates are reported per 100 000 men.

**Figure 3.**
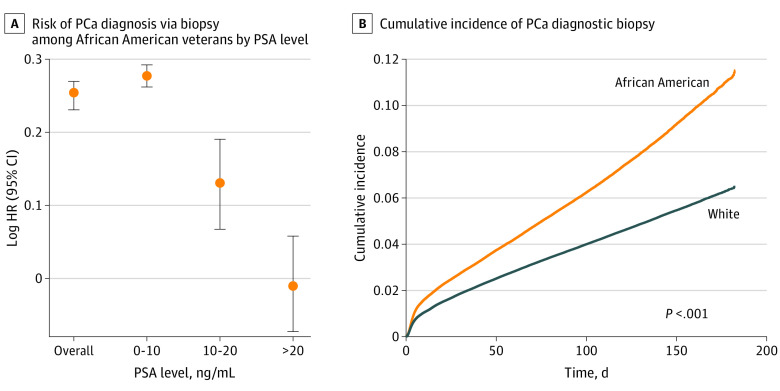
Time to Prostate Cancer (PCa) Diagnostic Biopsy Based on Screening Prostate-Specific Antigen (PSA) Level Among African American Veterans and Cumulative Incidence of PCa Diagnostic Biopsy A, Reference is White men. HR indicates hazard ratio.

Among 7 889 984 veterans (incidence cohort) undergoing routine care within the VA hospitals, age-adjusted rates of PCa were consistently higher among African American men than White men from 2005 through 2019 (Figure 2). In accordance with other population-level studies, African American men displayed a nearly 2-fold increased risk of developing PCa compared with White men within this network with relatively equal access to care (Figure 2A). Similarly, African American men had higher age-adjusted rates of de novo mPCa at diagnosis across all years (Figure 2B). We observed significant disparity in PCa incidence across the VA network, with African American veterans showing higher incidence of both localized PCa and de novo mPCa relative to White veterans (eFigure 1 in the Supplement; Figure 2C-E). Among veterans undergoing PSA screening (n = 2 788 003), African American men were more likely than White men to be diagnosed as having PCa , even after adjusting for PSA levels preceding biopsy (hazard ratio, 1.29; 95% CI, 1.27-1.31; *P* < .001). The risk of receiving a PCa diagnosis based on biopsy results was consistently higher among African American men with prebiopsy PSA levels lower than 10 ng/mL (hazard ratio, 1.32; 95% CI, 1.30-1.34; *P* < .001) and 10 to 20 ng/mL (hazard ratio, 1.14; 95% CI, 1.07-1.21; *P* < .001) (Figure 3A). African American men had an overall higher cumulative incidence of PCa based on diagnostic biopsy results compared with White men (Figure 3B).

### Treatment Delivery and Response in Relatively Equal-Access Settings

Overall follow-up in the analytical M0 cohort was relatively similar between African American veterans (median follow-up, 89 months [IQR, 60-122 months] and White veterans (median follow-up, 92 months [IQR, 60-125 months). African American veterans diagnosed as having localized PCa had a longer interval between diagnosis and primary treatment initiation than did White veterans. The overall median time to treatment was 125 days (IQR, 78-230 days) for African American men vs 110 days (IQR, 70-200 days) for White men (*P* < .001) (eTable 1 in the Supplement). African American men classified as “other” race within the treatment category had no documented primary treatment in the VA hospitals. This group displayed a significant increased risk (29%) of developing distant metastasis following diagnosis compared with White men (adjusted hazard ratio [aHR], 1.29; 95% CI, 1.17-1.42; *P* < .001) (Table). By contrast, among treated patients, African American men had an 11% lower risk of developing metastasis than White men (aHR, 0.89; 95% CI, 0.83-0.95; *P* < .001). Reduction in the risk of metastasis appears to be associated with radiation treatment because no race or ethnicity difference in the development of metastasis after treatment was observed in the prostatectomy group (aHR, 0.96; 95% CI, 0.86-1.08; *P* = .50) (Table). In the radiation group, the rate of distant metastasis was attenuated, with African American veterans experiencing lower risk of metastasis (aHR, 0.89; 95% CI, 0.83-0.97; *P* = .005). The cumulative incidence of metastasis at different time intervals is provided in eTable 2 in the Supplement. In subgroup analyses, we assessed our findings using the propensity score–matched M0 cohort. Results of the treatment-stratified subgroup analyses were consistent with our previous analyses, with African American men continuing to experience a lower risk of metastasis when treated at VA hospitals, whereas African American men in the “other” race treatment category remained at a higher risk of metastasis (eTable 3 in the Supplement). When we adjusted for the treatment type as a covariate, no racial and ethnic differences in the risk of metastasis were observed (eTable 4 in the Supplement).

**Table.** Time to Distant Metastasis by Treatment Among 92 269 Veterans Comprising the Localized Prostate Cancer (M0) Cohort

Variable	Other (n = 36 186)^a^		Primary treatment^b^					
			RT (n = 36 459)		RP (n = 19 624)		RT or RP (n = 56 083)	
	aHR (95% CI)	*P* value	aHR (95% CI)	*P* value	aHR (95% CI)	*P* value	aHR (95% CI)	*P* value
Race								
African American	1.29 (1.17-1.42)	<.001	0.89 (0.83-0.97)	.005	0.96 (0.86-1.08)	.50	0.89 (0.83-0.95)	<.001
White	1 [Reference]		1 [Reference]		1 [Reference]		1 [Reference]	
Age at diagnosis	0.999 (0.994-1.004)	0.60	1.001 (0.997-1.006)	.50	0.999 (0.991-1.007)	.70	0.996 (0.992-1.000)	.06
PSA level at diagnosis, ng/mL								
<10	1 [Reference]		1 [Reference]		1 [Reference]		1 [Reference]	
10-20	2.59 (2.29-2.92)	<.001	1.48 (1.34-1.63)	<.001	1.75 (1.54-2.00)	<.001	1.58 (1.46-1.71)	<.001
>20	6.34 (5.64-7.13)	<.001	3.08 (2.79-3.39)	<.001	2.24 (1.89-2.67)	<.001	2.77 (2.55-3.01)	<.001
Unknown	5.16 (3.04-8.76)	<.001	1.69 (0.84-3.39)	.10	0.93 (0.30-2.91)	.90	1.50 (0.83-2.72)	.10
Gleason score at diagnosis								
6	1 [Reference]		1 [Reference]		1 [Reference]		1 [Reference]	
7 (3 + 4)	1.77 (1.54-2.05)	<.001	1.48 (1.32-1.67)	<.001	1.68 (1.43-1.99)	<.001	1.57 (1.43-1.74)	<.001
7 (4 + 3)	2.85 (2.43-3.33)	<.001	2.25 (1.97-2.57)	<.001	2.56 (2.13-3.07)	<.001	2.41 (2.17-2.69)	<.001
≥8	5.35 (4.69-6.10)	<.001	3.85 (3.42-4.33)	<.001	4.63 (3.93-5.45)	<.001	4.21 (3.83-4.63)	<.001
Unknown	0.32 (0.24-0.42)	<.001	1.02 (0.61-1.71)	.90	0.35 (0.09-1.45)	.10	0.76 (0.47-1.24)	.20
ADT	NA	NA	1.49 (1.37-1.63)	<.001	4.79 (4.23-5.43)	<.001	1.76 (1.64-1.88)	<.001
Time to treatment, mo	NA	NA	1.012 (1.011-1.013)	<.001	0.999 (0.996-1.002)	.50	1.010 (1.008-1.011)	<.001
Diagnosis year	1.05 (1.03-1.07)	<.001	1.016 (1.002-1.031)	.02	1.07 (1.05-1.09)	<.001	1.03 (1.02-1.04)	<.001

Abbreviations: ADT, androgen deprivation therapy; aHR, adjusted hazard ratio; NA, not applicable; PSA, prostate-specific antigen; RP, radical prostatectomy; RT, radiotherapy.

^a^ Model to estimate the risk of metastasis in other treatment category (includes active surveillance, watchful waiting, cryotherapy, ADT only, or no treatment) was not adjusted for ADT use and time to treatment.

^b^ The HR estimates were not changed in sensitivity analyses when unknown PSA levels and Gleason scores were excluded.

**Figure 4.**
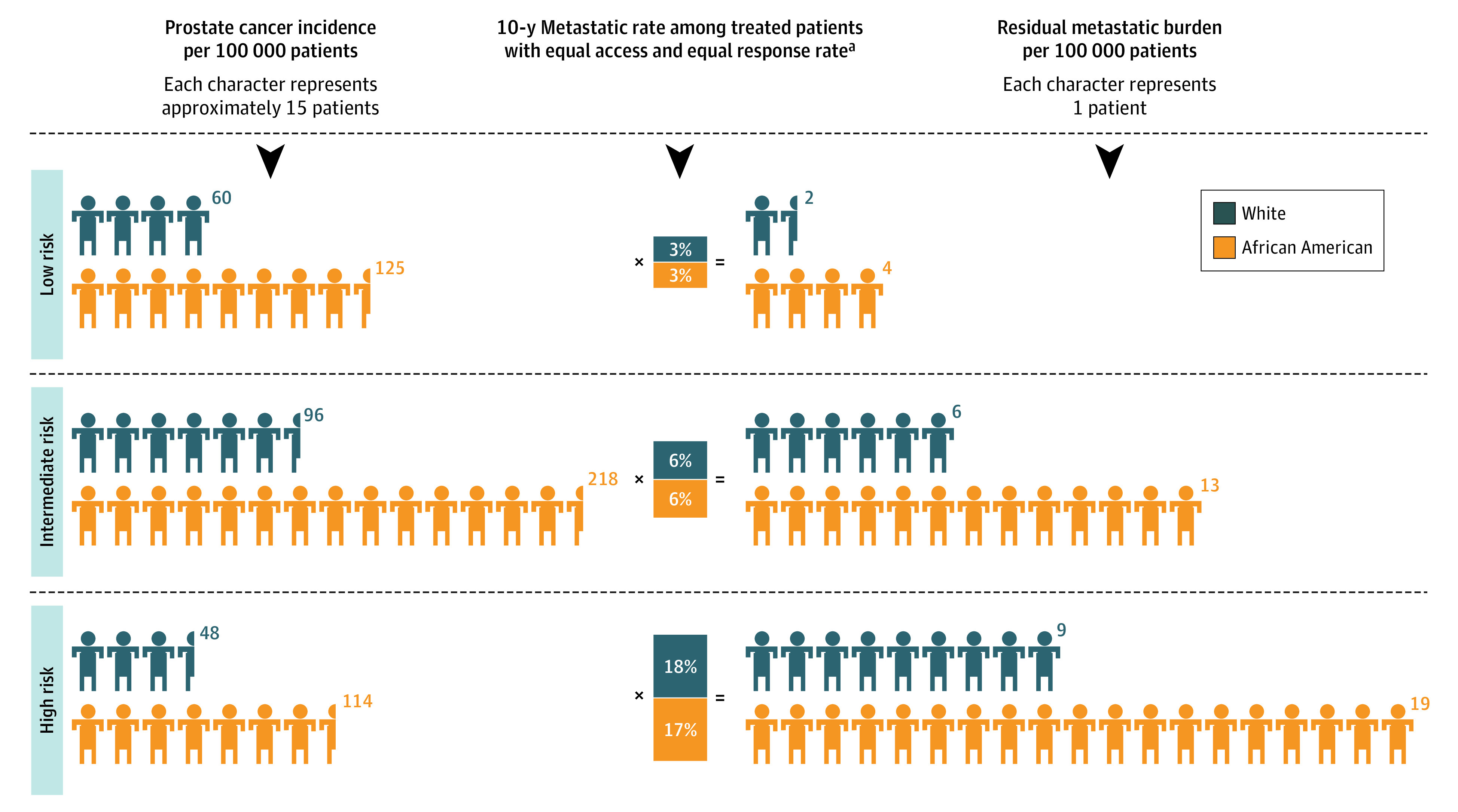
Residual Metastatic Burden Between African American and White Veterans Across National Comprehensive Cancer Network Risk Groups After Definitive Treatment Prostate cancer incidence rates across National Comprehensive Cancer Network risk groups are not age adjusted. ^a^Ten-year metastatic rates are derived by dividing the total number of metastatic events by the number of at-risk patients who received definitive primary treatment (n = 56 083).

Finally, we evaluated disparities in metastatic burden and PCSM among men at risk for PCa (Figure 4; eFigure 3 in the Supplement). For this residual metastatic burden, we estimated the rate of metastasis among 56 083 veterans who received definitive treatment and found that the proportions of metastatic cases were similar between African American men and White men. We derived the residual metastasis burden per 100 000 veterans by multiplying the proportions of metastatic cases by the NCCN risk–stratified incidence rates for African American men and White men. Despite equal response rates after treatment, a nearly 2-fold racial disparity in metastasis persisted across the NCCN risk groups between African American veterans and White veterans (low risk, 4 vs 2 per 100 000; intermediate risk, 13 vs 6 per 100 000; high risk, 19 vs 9 per 100 000) (Figure 4). Similar to metastatic disease, a residual burden of PCSM was evident for African American men compared with White men in the VA hospitals despite having equal treatment responses (eFigure 3 in the Supplement).

## Discussion

This cohort study extends previously published analyses of veterans with PCa that have tried to quantify PCa racial and ethnic disparities. In contrast to previously reported analyses, we analyzed electronic health record data across the PCa disease continuum, including incidence, clinical management, progression (distant metastasis), and survival outcomes. Considering the complex interplay among socioeconomic status and access to care, the environment, and lifestyles that likely influences tumor biology, our results provide a framework on which to evaluate how these factors are associated with incidence of disease and outcomes across self-identified racial and ethnic groups. A major finding of this work is the significantly higher incidence of both localized and de novo mPCa among African American veterans compared with White veterans. Although timely initiation of primary treatment was associated with a dissipation of several outcome-level disparities, factoring in the higher incidence rate was associated with increased metastasis rates persisting in the African American group.

At the population level, the incidence of PCa is historically higher among African American men (1 in 7) than among White (1 in 9) men.^1^ Overlaying these observed incidence-level differences with known PSA screening disparities suggests that the true incidence of PCa among African American men may be even higher than observed and reported.^19,20^ The VA eliminates many access-related barriers in PCa screening and provides an unbiased estimate of true incidence. We showed that a slightly higher proportion of African American veterans received diagnostic imaging, including prostate magnetic resonance imaging scans, as part of their initial workup compared with White veterans. However, despite similar access to screening protocols, a persistent disparity in the incidence PCa was observed among African American men for both localized and de novo mPCa. This disparity in the incidence of PCa was evident almost uniformly in a state-by-state analysis of VA hospitals across the network. The incidence of PCa among African American men did not appear to be associated solely with screening PSA levels because the likelihood of detecting incidental PCa after the initial biopsy remained higher across PSA level categories. Therefore, consistently higher incidence rates among African American men in an equal-access setting may suggest multifactorial etiologic factors, including neighborhood characteristics and environmental exposures, nutrition, life course stressors, and underlying ancestral or hereditary traits, that may predispose African American men to a higher risk of developing PCa. Emerging studies using similar equal-access data have not focused on the differential PCa incidence in their analysis.^5,8,21^ Consequently, this unaccounted-for disparity between African American men and White men persists even after achieving comparable outcomes after primary treatment, resulting in an increased metastatic burden and mortality over time. We showed this phenomenon by identifying metastatic and PCSM rates among African American veterans and White veterans who received primary curative-intent treatment of localized PCa. Indeed, following primary treatment, the outcomes were relatively similar between African American veterans and White veterans. However, when these outcomes were adjusted to account for the disparate incidence rates, we observed a persistent residual disparity in both metastasis and PCSM of approximately 2-fold among African American men compared with White men in the VA, which is comparable to the population-level disparity across the US.^1^

A collective body of evidence indicates a relatively similar risk of PCSM between African American men and White men whose cancer was staged according to NCCN criteria and who received curative-intent treatment in an equal-access setting.^5,6,8^ In fact, Rivieri and colleagues^8^ found that African American veterans had a lower risk of PCSM in this setting. Although our results were in line with previous VA-based studies, our study is unique in that we consider distant metastasis as a primary end point in lieu of long-term survival end points. A careful analytical interpretation of our results indicates that treatment may act as an equalizer of racial and ethnic disparities. The subset of patients treated within the VA hospitals responded favorably regardless of race and ethnicity and the NCCN risk category. Therefore, equitable and timely intervention can translate to improved outcomes, but it may not eliminate the residual disparity owing to incidence observed among African American men who continue to endure higher rates of distant metastasis despite receiving similar treatment.^5,6^ Therefore, a broader approach is warranted to understand the etiologic factors associatad with these racial and ethnic disparities.

The disparate risk of distant metastasis in African American men was significantly reduced when radiotherapy was the primary mode of curative-intent therapy. Consistent with prior population-level work by McKay et al,^6^ we showed that African American men respond more favorably to radiotherapy compared with White men. These observations may suggest the role of tumor biology because the enrichment of distinct genomic tumor subtypes within African American men may be associated with increased radiosensitivity.^22^ A growing body of evidence shows that certain features of tumors, such as radiosensitivity and an inflammatory tumor microenvironment, are enriched in men of African ancestry.^22,23^ In line with this hypothesis, recent studies have shown that prostate tumors from African American men manifest lower DNA damage response and may have increased susceptibility to DNA-damaging radiotherapy leading to a more favorable response to radiation-based treatments.^22,24^ These findings highlight the importance of studying the genomic diversity of prostate tumors among veterans who are more representative of the US population.

Overall, our work constitutes the largest study, to our knowledge, in an equal-access VA setting to evaluate the presence of racial and ethnic disparities across the PCa disease continuum, including nationwide incidence–level disparities. We also used the clinically robust end point of distant metastasis as an outcome.^11^ In addition, the use of nationwide VA incidence data enabled us to discern PCa burden within the VA system that is disproportionately associated with African American men despite removing the barriers to screening and access to treatment. Given that health care in an equal-access environment significantly diminishes social biases and inadequate access to screening and treatment options, the association of these barriers with care played a limited role in confounding the results presented herein. Therefore, our study presents an unbiased current state of racial and ethnic disparity at a national level.

### Limitations

The limitations of this study include how findings within the VA population translate to academic and community-based health care populations. In addition, the VA Informatics and Computing Infrastructure interface is a complex integration of data from different sources; therefore, missing data and data quality may have affected the analysis.

## Conclusions

This cohort study found that African American men with access to VA health care equal to White men had a higher burden of PCa in terms of incidence, morbidity, and mortality. Although equitable treatment has the potential to attenuate disparity gaps in outcomes, it did not completely eliminate the residual disparity associated with the increased incidence of PCa among African American men.

## Notes

Supplement. **eTable 1.** Characteristics of the Localized (M0) PCa Cohort **eTable 2.** Cumulative Incidence of Metastasis at 1, 5, and 10 Years Between Racial Groups Stratified By Treatment Types **eTable 3.** Risk of Metastasis in the Propensity Matched Cohort **eTable 4.** Estimated Risk of Metastasis by Racial Group in the Treatment-Adjusted Model **eFigure 1.** Estimation of Nationwide Racial Disparities in the VA **eFigure 2.** Likelihood of Having High Gleason Score (≥4+3), PSA (≥20 ng/ml) and High-Risk PCa (PSA>20 or Gleason score ≥8 or T3 disease and above) in African American Veterans **eFigure 3.** Residual PCSM Burden Between African American (AA) and White (ie, European American [EA]) Veterans Across NCCN Risk Groups After Definitive Treatment
