# Errors in Abstract and Results

**DOI:** 10.1001/jamanetworkopen.2022.2773

**Published:** 2022-02-21

In the Original Investigation titled “Racial and Ethnic Disparities in Prostate Cancer Outcomes in the Veterans Affairs Health Care System,”^1^ published January 18, 2022, there were errors in the Results section of the abstract and the Results. In the abstract, African American men who were classified as “other” race and received treatment should have been described as African American men who received nondefinitive treatment classified as “other.” In the Results, “African American men classified as ‘other’ race within the treatment category had no documented primary treatment in the VA hospitals” should be changed to “Veterans who had no documented primary treatment in the VA hospitals or received nondefinitive treatment were classified as ‘other.’” The African American men in this group displayed a significant increased risk of developing distant metastasis following diagnosis compared with White men. This article has been corrected.^1^
