# Supplementary material for: Racial and Ethnic Disparities in Prostate Cancer Outcomes in the Veterans Affairs Health Care System
Source: JAMA Netw Open. 2022 Jan 18;5(1):e2144027. doi: 10.1001/jamanetworkopen.2021.44027 (PMC8767437; doi:10.1001/jamanetworkopen.2021.44027)
Supplement: Supplement. — eTable 1. Characteristics of the Localized (M0) PCa Cohort eTable 2. Cumulative Incidence of Metastasis at 1, 5, and 10 Years Between Racial Groups Stratified By Treatment Types eTable 3. Risk of Metastasis in the Propensity Matched Cohort eTable 4. Estimated Risk of Metastasis by Racial Group in the Treatment-Adjusted Model eFigure 1. Estimation of Nationwide Racial Disparities in the VA eFigure 2. Likelihood of Having High Gleason Score (≥4+3), PSA (≥20 ng/ml) and High-Risk PCa (PSA>20 or Gleason score ≥8 or T3 disease and above) in African American Veterans eFigure 3. Residual PCSM Burden Between African American (AA) and White (ie, European American [EA]) Veterans Across NCCN Risk Groups After Definitive Treatment [file jamanetwopen-e2144027-s001.pdf]

Supplementary Online Content

Yamoah K, Lee KM, Awasthi S, et al. Racial and ethnic disparities in prostate cancer outcomes in the Veterans Affairs health care system. *JAMA Netw Open*. 2022;5(1):e2144027. doi:10.1001/jamanetworkopen.2021.44027

eTable 1. Characteristics of the Localized (M0) PCa Cohort

eTable 2. Cumulative Incidence of Metastasis at 1, 5, and 10 Years Between Racial Groups Stratified By Treatment Types

eTable 3. Risk of Metastasis in the Propensity Matched Cohort

eTable 4. Estimated Risk of Metastasis by Racial Group in the Treatment-Adjusted Model

eFigure 1. Estimation of Nationwide Racial Disparities in the VA

eFigure 2. Likelihood of Having High Gleason Score (≥4+3), PSA (≥20 ng/ml) and High-Risk PCa (PSA>20 or Gleason score ≥8 or T3 disease and above) in African American Veterans

eFigure 3. Residual PCSM Burden Between African American (AA) and White (ie, European American [EA]) Veterans Across NCCN Risk Groups After Definitive Treatment

This supplementary material has been provided by the authors to give readers additional information about their work.

© 2022 Yamoah K et al. *JAMA Network Open*.

eTable 1. Characteristics of the Localized (M0) PCa Cohort

n = 92,269

**Variables**	**AA**/Black **(n = 28,802)**	**White (n = 63,467)**	**p**
**Number of Biopsies[1]**			
Mean (Range)	1.2 (5)	1.2 (6)	0.5
**Time from first PSA to Dx (Days)**			
Median (IQR)	2,125 (2,447.5)	1,897 (2,288)	<0.0001
**Age at Dx**			
Median (IQR)	56 (11)	61 (9)	<0.0001
**Imaging[2]**	15,114 (52%)	30,002 (47%)	<0.0001
**MRI (Prostate/Pelvis)[3]**	986 (3%)	1562 (2%)	<0.0001
**Gleason Score**			<0.0001
6	10,876 (38%)	25,302 (40%)	
7 (3+4)	8,310 (29%)	16,195 (26%)	
7 (4+3)	3,432 (12%)	6,838 (11%)	
≥ 8	4,285 (15%)	9,658 (15%)	
Unk	1,899 (7%)	5,474 (9%)	
**PSA**			<0.0001
<10	21,142 (73%)	50,650 (80%)	
10 to 20	4,705 (16%)	8,658 (14%)	
>20	2,908 (10%)	4,005 (6%)	
Unk	43 (0%)	154 (0%)	
**Time to Treatment (Days)**			
RT (Median, IQR)	132 (177)	114 (155)	< 0.0001
RP (Median, IQR)	112 (110)	104 (94)	
Overall	125 (152)	110 (130)	

Abbreviations: AA, African American; Dx, Diagnosis; IQR, Interquartile range; MRI; Magnetic resonance imaging; PSA, Prostate Specific Antigen; NCCN, National Comprehensive Cancer Network; RT, Radiotherapy; RP, Radical Prostatectomy.

[1]Number of Biopsies prior to having a positive (diagnostic) biopsy

[2]Any imaging (CT, MRI, Bone Scans) within a year of having diagnostic biopsy

[3]MRI prostate/pelvis use during year of dx bx

© 2022 Yamoah K et al. *JAMA Network Open*.

eTable 2. Cumulative Incidence of Metastasis at 1, 5, and 10 Years Between Racial Groups Stratified By Treatment Types

	**Time duration**		
**Race Group**	**1 year**	**5 year**	**10 Year**
**Treatment Type = Other**			
AA	0.01	0.05	0.10
White	0.01	0.04	0.06
**Treatment Type = RT**			
AA	0.009	0.05	0.10
White	0.01	0.05	0.10
**Treatment Type = RP**			
AA	0.02	0.05	0.08
White	0.02	0.05	0.08
Cumulative incidences are reported as proportion			

© 2022 Yamoah K et al. *JAMA Network Open*.

eTable 3. Risk of Metastasis in the Propensity Matched Cohort

n = 57,604

	**Other (n=19,577)***				**Primary treatment = RT (n=24,519)**				**Primary treatment = RP (n=13,508)**				**Primary treatment = RT or RP (n=38,027)**			
	**HR**	**CI**		**p**	**HR**	**CI**		**p**	**HR**	**CI**		**p**	**HR**	**CI**		**p**
**Race**																
White	**1(ref)**				**1(ref)**				**1(ref)**				**1(ref)**			
AA/Black	1.35	1.21	1.51	< 0.01	0.86	0.79	0.94	< 0.01	0.95	0.84	1.08	0.45	0.87	0.81	0.93	< 0.01
**ADT**	-	-	-	-	2.99	2.73	3.28	< 0.01	8.64	7.57	9.86	< 0.01	3.44	3.21	3.70	< 0.01
**Months to Tx**	-	-	-	-	1.008	1.006	1.009	< 0.01	0.99	0.98	0.99	< 0.01	1.005	1.003	1.006	< 0.01
**Dx year**	1.001	0.98	1.02	0.95	1.03	1.02	1.05	< 0.01	1.10	1.07	1.12	< 0.01	1.05	1.04	1.07	< 0.01

Abbreviations: AA, African American; Dx, Diagnosis, Tx, Treatment; RT, Radiation therapy, RP, Radical Prostatectomy
*Model to estimate the risk of metastasis in other treatment category (Includes active surveillance, watchful waiting, cryotherapy, androgen deprivation therapy only, or no treatment) was not adjusted for ADT use and time to treatment. Data used in this subgroup validation analysis was matched between AA/Black (n = 28,802) and White (n = 28,802), by age, PSA and Gleason score

© 2022 Yamoah K et al. *JAMA Network Open*.

eTable 4. eEstimated Risk of Metastasis by Racial Group in the Treatment-Adjusted Model

n = 57,604

**Race Group**	**Hazard Ratio**	**95% CI**		**p**
White	1 (ref)			
AA/Black	1.05	0.99	1.11	0.12
**Abbreviations:** AA, African American; CI, Confidence interval.				
Model is adjusted by using treatment type as a confounder				
Data used in this subgroup validation analysis was matched between AA/Black (n = 28,802) and White (n = 28,802), by age, PSA and Gleason score				

© 2022 Yamoah K et al. *JAMA Network Open*.

eFigure 1. Estimation of Nationwide Racial Disparities in the VA

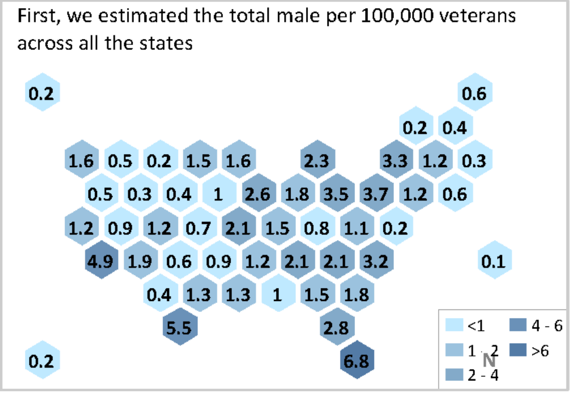

1. **Localized PCa incidence stratified by AA and EA.**

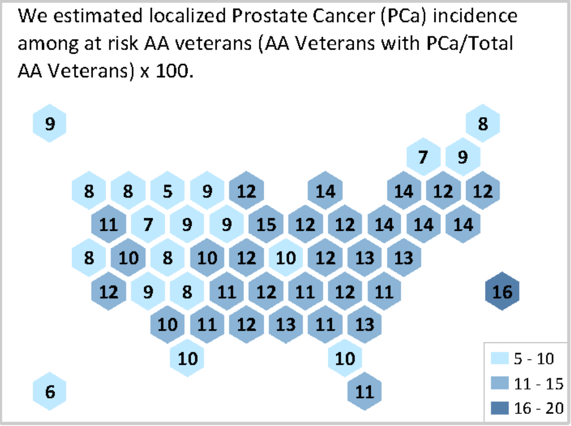

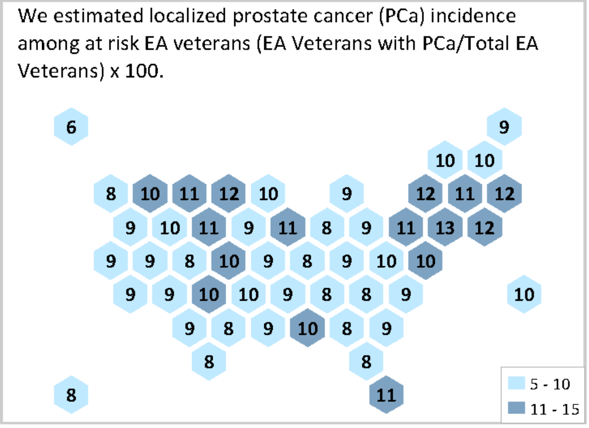

Using localized PCa incidence estimates we calculated localized PCa incidence in AA relative to EA (Figure 2D Localized PCa).

2. ***De novo* metastasis incidence stratified by AA and EA**

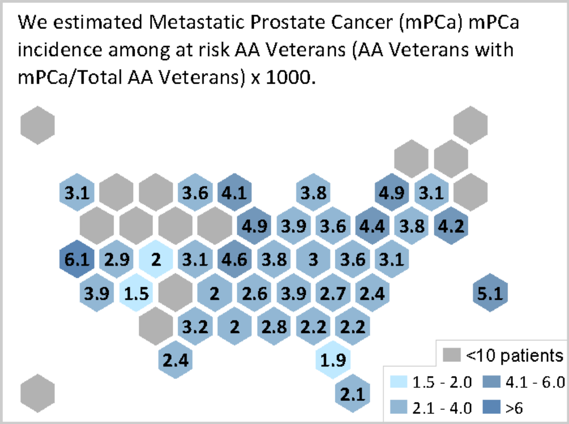

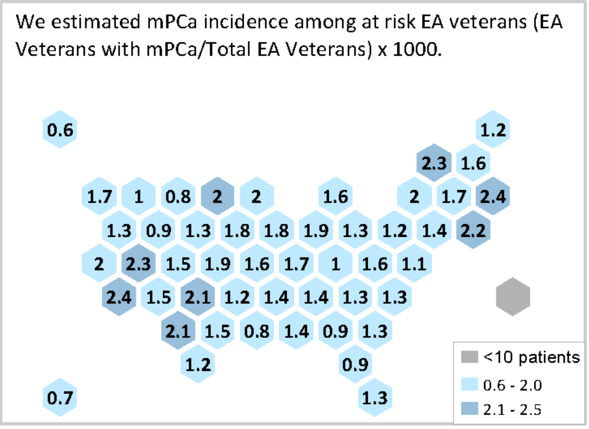

Using mPCa estimates we calculated mPCa Incidence in AA relative to EA (Figure 2E *De novo* metastasis).

© 2022 Yamoah K et al. *JAMA Network Open*.

eFigure 2. Likelihood of Having High Gleason Score (≥4+3), PSA (≥20 ng/ml) and High-Risk PCa (PSA>20 or Gleason score ≥8 or T3 disease and above) in African American Veterans

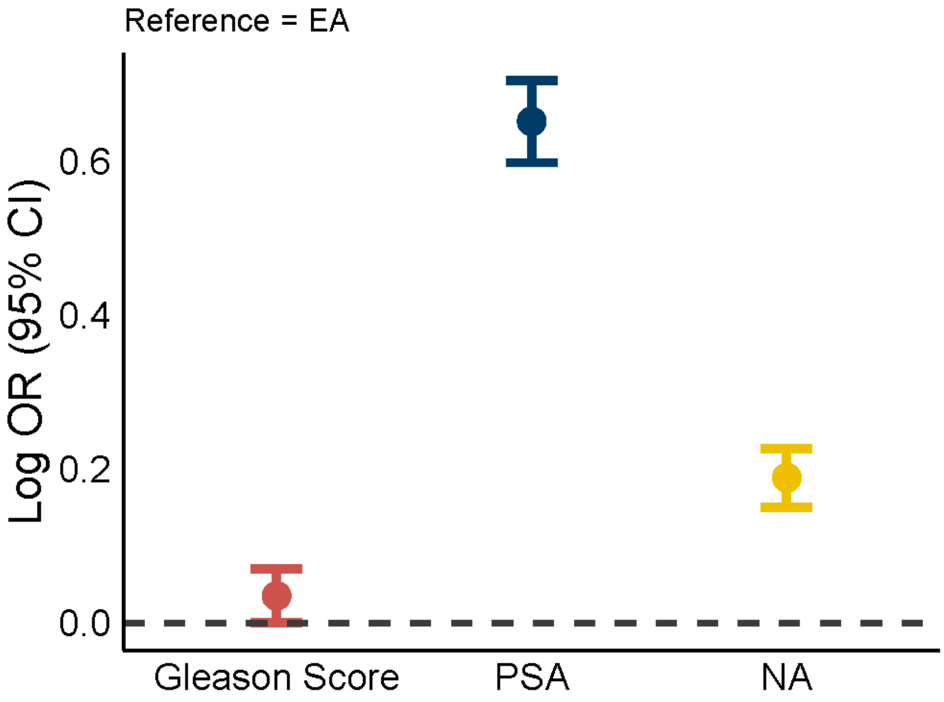

© 2022 Yamoah K et al. *JAMA Network Open*.

eFigure 3. Residual PCSM Burden Between African American (AA) and White (ie, European American [EA]) Veterans Across NCCN Risk Groups After Definitive Treatment

Prostate cancer incidence rates across NCCN risk groups are not age adjusted.

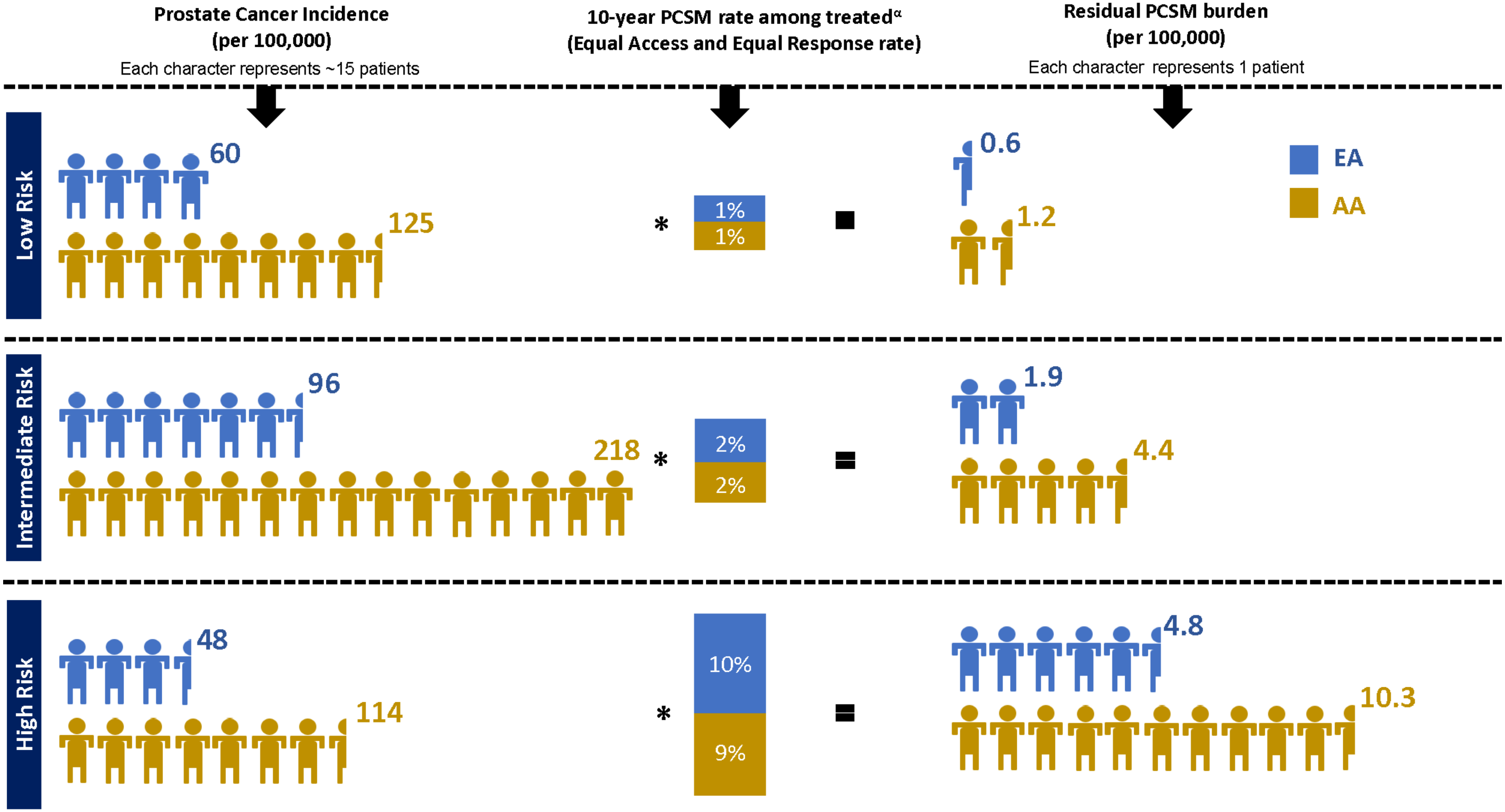

[α] 10-year PCSM rates are derived by dividing the total number of PCSM event by the number of at-risk patient who received definitive primary treatment (n = 56,083)

© 2022 Yamoah K et al. *JAMA Network Open*.
